# Development of a new Emergency Medicine Spinal Immobilization Protocol for trauma patients and a test of applicability by German emergency care providers

**DOI:** 10.1186/s13049-016-0267-7

**Published:** 2016-05-14

**Authors:** Michael Kreinest, Bernhard Gliwitzky, Svenja Schüler, Paul A. Grützner, Matthias Münzberg

**Affiliations:** BG Trauma Center Ludwigshafen, Department of Trauma Surgery and Orthopaedics, Ludwig-Guttmann-Str. 13, 67071 Ludwigshafen, Germany; PHTLS Europe Research Group, Offenbach/Queich, Germany; University of Heidelberg, Institute for Medical Biometry and Informatics, Heidelberg, Germany

**Keywords:** Out-of-hospital, Emergency, Cervical, Spine, Protocol, Algorithm, Immobilization

## Abstract

**Background:**

In order to match the challenges of quickly recognizing and treating any life-threatening injuries, the ABCDE principles were established for the assessment and treatment of trauma patients. The high priority of spine protection is emphasized by the fact that immobilization of the cervical spine is performed at the very first step in the ABCDE principles. Immobilization is typically performed to prevent or minimize secondary damage to the spinal cord if instability of the spinal column is suspected. Due to increasing reports about disadvantages of spinal immobilization, the indications for performing spinal immobilization must be refined.

The aim of this study was (i) to develop a protocol that supports decision-making for spinal immobilization in adult trauma patients and (ii) to carry out the first applicability test by emergency medical personnel.

**Methods:**

A structured literature search considering the literature from 1980 to 2014 was performed. Based on this literature and on the current guidelines, a new protocol that supports on scene decision-making for spinal immobilization has been developed. Parameters found in the literature concerning mechanisms and factors increasing the likelihood of spinal injury have been included in the new protocol. In order to test the applicability of the new protocol two surveys were performed on German emergency care providers by means of a questionnaire focused on correct decision-making if applying the protocol.

**Results:**

Based on the current literature and guidelines, the Emergency Medicine Spinal Immobilization Protocol (E.M.S. IMMO Protocol) for adult trauma patients was developed. Following a fist applicability test involving 21 participants, the first version of the E.M.S. IMMO Protocol has to be graphically re-organized. A second applicability test comprised 50 participants with the current version of the protocol confirmed good applicability. Questions regarding immobilization of trauma patients could be answered properly using the E.M.S. IMMO Protocol.

**Discussion:**

Current literature increasingly reports of disadvantages that may be associated with immobilization. Based on the requirements of the current guidelines, a new protocol that supports decision-making for indications for out-of-hospital spinal immobilization has been developed in this study. In contrast to established protocols, the new protocol offers different options for immobilization as well as a decicion-support.

**Conclusions:**

The E.M.S. IMMO protocol provides a decision-support tool for indications for spinal immobilization in adult trauma patients that permits variable decision-making depending on the current condition of the trauma patient and the pattern of injuries for immobilization in general and for immobilization method in particular.

## Background

Rapid recognition of any life-threatening injury as well as fast prioritization of treatments that are immediately required may be considered the greatest challenges facing emergency medical personnel in treating trauma patients [1]. To match these challenges, treatment of severely injured patients should follow a structured protocol [2]. Treatment of trauma patients commonly follows the ABCDE concept that provides a clear basis for prioritization (Table 1) [2].

**Table 1:** The ABCDE concept for treating trauma patients

A	Airway/Cervical Spine Protection
B	Breathing
C	Circulation
D	Disability
E	Exposure/Environment

The importance of protecting the spine is emphasized by the fact that immobilization of the cervical spine is performed at the very first step of the ABCDE principles (Table 1). Spinal immobilization is performed primarily to prevent or minimize secondary damage to the spinal cord caused by injuries causing instability of the spinal column [3]. Although previous studies have attributed the frequently observed prehospital neurological deterioration [3] in patients with spinal injuries to the failure of immobilizing the spine [4, 5], more recent work has not confirmed this relationship [6, 7]. Today, it is generally recognized that there is no clear evidence either for or against immobilization [7–10]. Indeed, it is increasingly more important to accept that both the use of a cervical collar and full body immobilization are associated with disadvantages. First, use of a cervical collar on its own does not provide full immobilization of the cervical spine, as there is still considerable residual mobility. This residual mobility is evident in all models of cervical collars that have been tested [11–13]. Thus, to protect the spinal column, full immobilization of head and trunk is necessary [14–16]. Moreover, a cervical collar can result in compression of the jugular veins [17] and hence can lead to a significant increase in intracranial pressure [18–22]. Even full body immobilization, for example on a spine board, is not without complications. In healthy young subjects, complete immobilization was associated with restrictive effects on pulmonary function [23]. In general, airway management is impeded in immobilized patients [24, 25]. Immobilization on a spine board may also cause pain [26–28] and may result in pressure ulcers [29].

Immobilization of the spine in general and of the cervical spine in particular has been a standard procedure in prehospital treatment of trauma patients for many decades [8, 9]. However, due to the potential for complications cited above, spinal immobilization should not be performed on a routine basis but only if there are given indications, as it is also required by current guidelines [30–33]. As the severity of patient’s injuries increases, the likelihood of an associated spinal injury also increases [34]. However, clear prioritization of all procedures is especially imperative in such patients, as full immobilization of trauma patients can also be associated with delays [35] and even increase mortality, for example in patients with penetrating trauma [36]. Therefore, it is to question if severly injured patients should always be immobilized even if they have a higher probability of spinal injury. The guidelines recommend the use of a decision-support tool to facilitate a rapid and valid on scene decision [30, 31, 33].

A number of decision-support tools have been described in the literature. Some were initially designed for indications for radiological imaging in emergency rooms and were later on tested for sensitivity and specificity concerning the indications for prehospital spinal immobilization [37]. Applicability of many decision-support tools is subject to many limitations. For example, the majority of decision tools were developed for conscious and oriented patients [38, 39]. Many decision tools preclude penetrating [40–42] or blunt injuries [43]. Often, the current status of the patient (stable or unstable) is not considered [39, 41, 44] or the decision tool is only applicable if circulation is already stabilized [38]. Other decision-support tools are designed specifically for victims of motorcycle accidents [45] and not for general use. To our knowledge, there is no universally applicable protocol that supports decision-making for indications for spinal immobilization being valid for all adult trauma patients including the severly injured with unstable condition.

The aim of this study was (i) to develop a protocol as a decision-tool for indications for spinal immobilization in adult trauma patients and (ii) to carry out the first applicability tests of this protocol by emergency medical personnel by means of a questionnaire. The decision-support tool should be based on current literature and should orientate on the established ABCDE principles of trauma care. The differentiated consideration of various methods of immobilization and their potentials for complication should be taken into account as well as the patient’s condition into the new Emergency Medicine Spinal Immobilisation (E.M.S. IMMO) Protocol.

## Methods

The current study has been approved by the ethical committee in charge (Ethics committee of the State Medical Association Rhineland-Palatinate, Mainz, Germany) under the reference number 837.371.13 (9056).

### Development of the E.M.S. IMMO Protocol

A structured search of the United States National Library of Medicine and the National Institutes of Health database was performed using MEDLINE through PubMed (www.pubmed.gov). The search terms used are listed in Table 2. We considered the literature from 1980 to 2014. Only articles written in english or german were viewed. Additional articles listed in the reference sections of these articles were also included. Original articles as well as review articles and articles about current guidelines were included. All articels found by the literature search were read full text by the authors.

**Table 2:** Search terms utilized in PubMed

Cervical spine immobilization	
Cervical spine immobilisation	
Spine AND motion	
Spine AND protocol	
Spine AND ((prehospital) OR (out-of-hospital) OR)	
(Spine AND injury) AND ((prehospital) OR (out-of-hospital))	
(Spine immobilization) AND ((prehospital) OR (out-of-hospital))	
(Spine immobilisation) AND ((prehospital) OR (out-of-hospital))	
((Spine injury) OR (spine trauma)) AND ((prehospital) OR (out-of-hospital))	

A new protocol that supports on scene decision-making for indications for spinal immobilization was developed based on the protocols found in the literature and taking into account the mechanisms and factors that increase the likelihood of spinal injury in an accident. Most of the parameters found in the literature were included in the new protocol. If contrary statements about spinal immobilization were found in the literature, the more scientifically substantiated statement was included in the current protocol. These decisions are discussed in detail.

### Testing the applicability of the E.M.S. IMMO Protocol

We surveyed German emergency rescue personnel and German emergency doctors in order to test the applicability of the new protocol for indications for spinal immobilization. The questionnaire included four questions about the E.M.S. IMMO Protocol (Fig. 3). After this survey, the protocol had to be revised based on the results of the questionnaire. After the revision, the new protocol was assessed again by the same questionnaire by German emergency personnel and German emergency doctors (not including the former participants). The questions of the first and second evaluations were identical (Fig. 3). All questions were evaluated using a scale of 1–6 (1 = “I agree completely”, 6 = “I disagree completely”).

### Statistics

Only fully completed questionnaires were included in the analysis. All data were evaluated at a descriptive level (median, interquartile range) and are presented as boxplots with outliers. The analysis was performed using SPSS for Windows, Version 22.0.

## Results

### Literature search

The literature search, carried out as described above, yielded 162 articles after removing duplicates. A reference analysis found additional 34 articles. Thus, a total of 196 articles were included and read in full text. The key conclusions were summarized in table format.

### Development of the E.M.S. IMMO Protocol

**Fig. 1:**
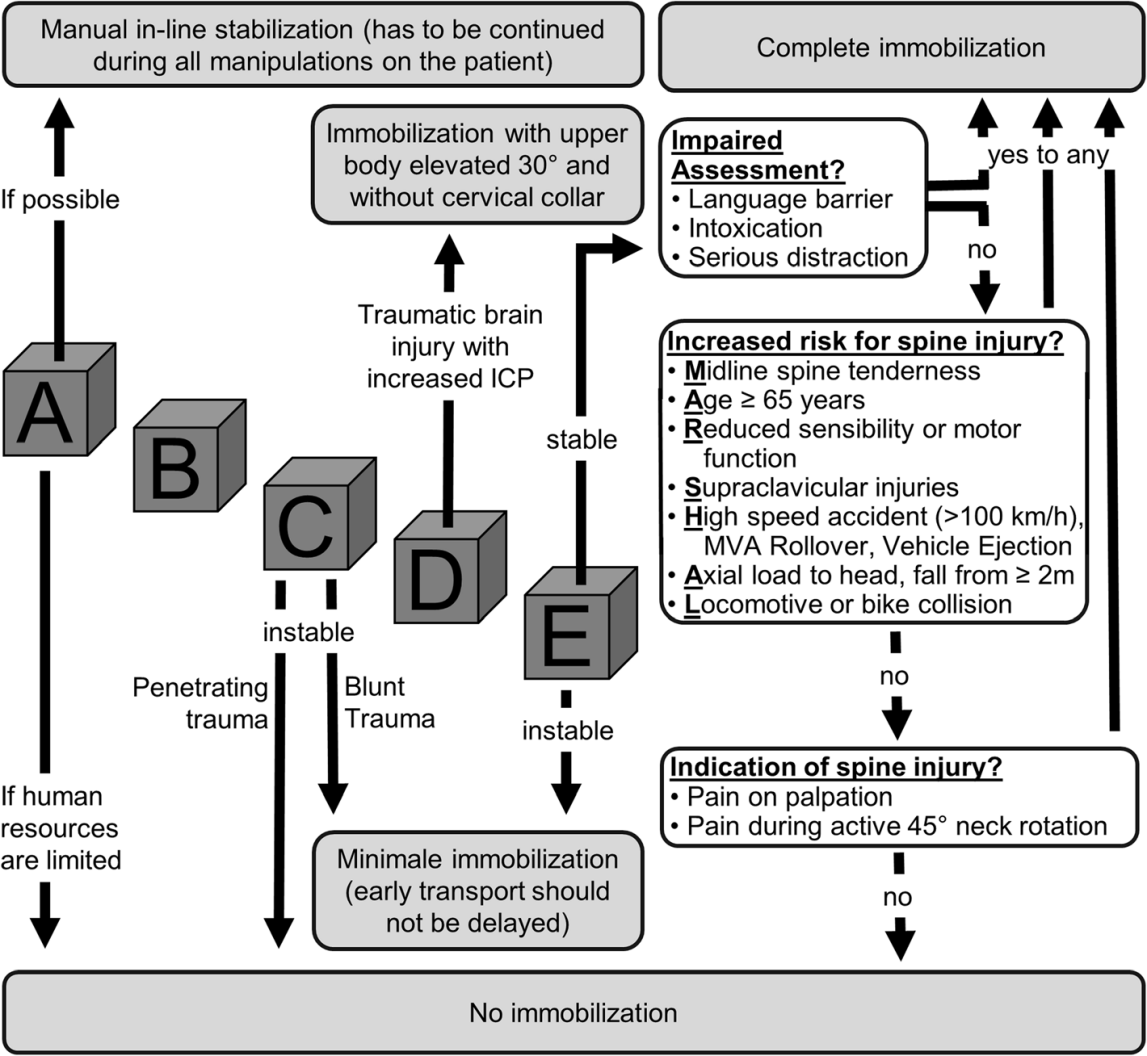
E.M.S. IMMO Protocol for adult trauma patients. The ABCDE concept is a central element of the protocol. Depending on the status of the patient, differentiated indications for various options for spinal immobilization are followed. For stable patients, the indication is based on the MARSHAL criteria and examination of the cervical spine, after the assessment of the patient has been evaluated as appropriate (ICP = intracranial pressure)

Since treatment of trauma patients according to ABCDE principles (Table 1) is already established in resuscitation room care (e. g. Advanced Trauma Life Support) and in prehospital treatment (e. g. Pre Hospital Trauma Life Support), the E.M.S. IMMO Protocol was also based on the ABCDE principle (Fig. 1).

**Fig. 2:**
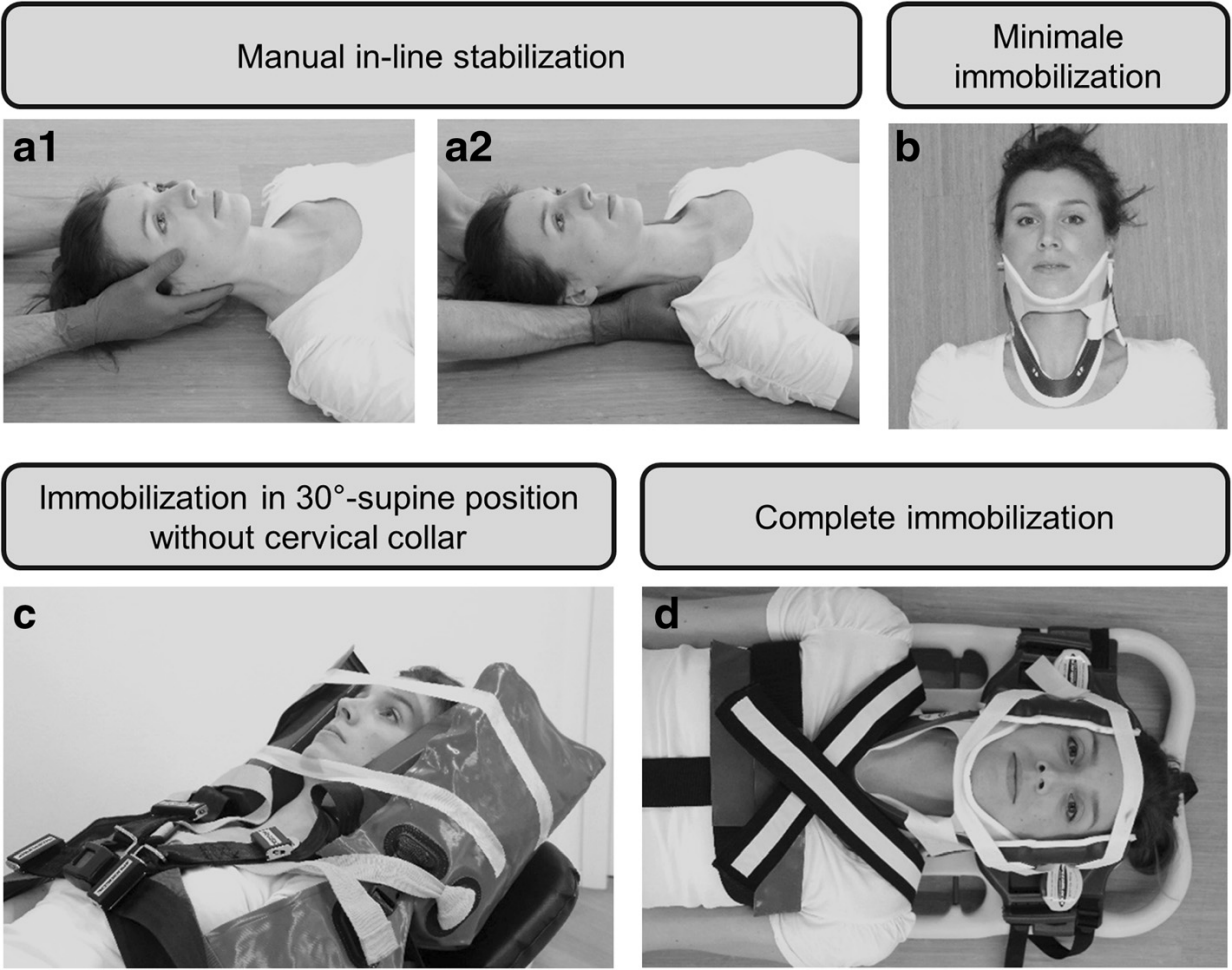
The different types of immobilization using the E.M.S. IMMO Protocol. Every trauma patient should first be stabilized using manual immobilization of the cervical spine (**a**). Patients who are unstable and with high transport priority should receive only minimal immobilization using a cervical collar (**b**). Immobilization of patients who show signs of increased intracranial pressure is achieved in the vacuum mattress in a 30° position with elevated upper body and no cervical collar (**c**). Complete immobilization may be indicated for patients who are haemodynamically stable (**d**). [Note: The model used in these photographs gave her permission for publication]

If possible, immobilization of the cervical spine should always take place immediadetly at initial contact to the trauma patient [2, 31]. In order to avoid delays caused by positioning a cervical collar prior to assessing the patient following the ABCDE principles, immobilization can be achieved by restraining the head using hands (Fig. 2a1) or forearms (Fig. 2a2) [46]. The so-called manual in-line stabilization is maintained throughout the ABCDE assessment and treatment of the trauma patient. Whenever possible, all procedures performed on a trauma patient (e. g. airway management, turning maneuvers, etc.) should be performed with a minimum of further manipulation of the spine in general and in the cervical spine in particular.

Trauma patients should be placed supine for initial assessment and further treatment while manual in-line stabilization is maintained. The cervical spine should be placed in neutral position.

If the assessment of a trauma patient indicates unstable circulation conditions (C according to the ABCDE principles, Table 1), priority for transportation is high. In the event of blunt trauma, immobilization may be minimized using just a cervical collar (Fig. 1). Although the use of a cervical collar alone (Fig. 2b) does not adequately restrict the mobility of the cervical spine [14–16], the residual mobility is accepted in this case, taking into account that complete immobilization would delay expeditious transport and thus could lead to increased mortality. According to the literature, trauma patients with unstable circulation following penetrating trauma should not be immobilized (Fig. 1), because the benefits are regarded as highly questionable [33, 36, 43, 47, 48]. Assessment of a trauma patient for neurological deficit (D, Table 1) should include determining if there are signs of severe brain injury or craniocrebral trauma with increased intracranial pressure. If there are signs of increased intracranial pressure (Table 3), the E.M.S. IMMO Protocol recommends that no cervical collar is used (Fig. 1), as this may cause further significant increases to intracranial pressure [20–22]. However, because cervical spine injuries often coincide with craniocerebral trauma [34], immobilization using the vacuum mattress is recommended [31]. Even without a cervical collar, modern vacuum mattresses could prove to achieve good immobilization (Fig. 2c). Moreover, a position with the upper body elevated 30° is possible (Fig. 2c), which is also recommended for patients with craniocerebral trauma [31]. Alternatively, the patient may be immobilized on a spine board, with which it has been show that complete immobilization is possible even without the use of a cervical collar [25].

**Table 3:** Evidence of an increase in intracranial pressure following craniocerebral trauma

Possible indications of increased intracraniall pressure:	Reduced vigilance
	Drop on Glasgow coma scale by ≥ 2
	Delayed pupil response
	Development of hemiparesis
Definitive indications of increased intracranial pressure:	Both pupils dilated
	Anisocoria and reduced vigilance
	Bending and stretching synergisms
	Cushing‘s triad
	- Hypertension
	- Bradycardia
	- Pathological breathing pattern

Patients who initially are in a stable circulation condition and no indications of increased intracranial pressure are obvious should have outer clothing removed and should then being examined in more detail (E, Table 1). Afterwards, a decision about transport priority has to be made. Therefore, a review of whether the patient’s condition is acute and life-threatening should be made. If so, there is high priority for transport and again only minimal immobilization of the cervical spine is performed using a cervical collar (Figs. 1 and 2b) for the reasons described above. If the patient is in a stable condition, it is necessary to assess the indications for full body immobilization, for example using a spine board (Fig. 2d) or vacuum mattress or whether the manual immobilization (Fig. 2a) that was maintained up to this point can be anulled. Criteria based on the literature were developed to support decision-making either for or against spinal immobilization. Table 4 provides an overview of the criteria found in the literature and indicates whether they were integrated into the E.M.S. IMMO Protocol.

**Table 4:** Criteria PRO and CONTRA spinal immobilization taken from literature search and integration into the E.M.S. IMMO Protocol

PRO criteria	References	E.M.S. IMMO protocol
Age > 65 years	[4, 21, 43]	included in MARSHAL criteria
Rigid vertebral disease	[52]	not included as PRO criteria
State of acute anxiety	[53]	included in MARSHAL criteria (serious distraction)
Language barrier	[53]	included in assessment of impairment
Acute stress reaction	[54]	included in MARSHAL criteria (serious distraction)
Distracting injury	[42, 44, 54]	included in MARSHAL criteria (serious distraction)
Intoxication	[52, 54–56]	included in assessment of impairment
Fall from > 6 m	[57]	included in MARSHAL criteria (fall from ≥ 2 m)
Fall from 3 to 6 m	[57]	included in MARSHAL criteria (fall from ≥ 2 m)
Fall from > 3 m	[52, 58]	included in MARSHAL criteria (fall from ≥ 2 m)
Fall from > 2 m	[59]	included in MARSHAL criteria (fall from ≥ 2 m)
Fall from > 1 m	[38, 44]	not included as PRO criteria
Fall from large animal	[57]	included in MARSHAL criteria (fall from ≥ 2 m)
High speed accident > 100 km/h	[38, 44, 52]	included in MARSHAL criteria
Speed > 56 km/h	[58]	not included as PRO criteria
MVA or pedestrian vs. train	[57]	included in MARSHAL criteria (locomotive or bike collision)
MVA ejection	[38, 44, 57]	included in MARSHAL criteria
Vehicle rollover	[38, 44]	included in MARSHAL criteria
Bicycle collision	[38, 44]	included in MARSHAL criteria
Road traffic collision	[59]	not included as PRO criteria
Significant intrusion of vehicle	[44]	not included as PRO criteria
Axial load to head	[38, 44]	included in MARSHAL criteria
Diving accident	[52]	included in MARSHAL criteria (axial load to head)
Sport injuries	[59]	not included as PRO criteria
Shooting	[59]	not included as PRO criteria
Death at scene	[58, 60]	not included as PRO criteria
Altered/loss of consciousness	[52–56, 61, 62]	included in ABCDE criteria (unstable patient)
Spine pain/tenderness	[4, 11, 20, 21, 24, 41, 49]	included in MARSHAL criteria and in indication of spine injury
Abnormal sensory/motor exam	[44, 45, 52, 55, 56, 62]	included in MARSHAL criteria
Significant head or facial injury	[52, 58, 61]	included in MARSHAL criteria (supraclavicular injuries)
Other spine fractures	[52]	not included as PRO criteria
Supraclavicular lesions	[45]	included in MARSHAL criteria
Severe injuries to other body systems	[52, 55, 56, 58, 61]	not included as PRO criteria
CONTRA criteria	References	E.M.S. IMMO protocol
No neurological abnormalities	[39, 50]	included in MARSHAL criteria
No evidence of intoxication	[39, 50, 63]	included in assessment of impairment
No midline C-spine tenderness	[39, 50]	included in MARSHAL criteria
No distracting injury	[39, 50, 63]	included in assessment of impairment
Able to actively rotate neck	[38, 44]	included in indication of spine injury
Penetrating trauma	[33]	not included as CONTRA criteria
Functional range-of-motion	[64]	included in indication of spine injury

The first assessment to be made is whether the stable patient can be adequately assesed (Fig. 1). An adequate assessment is not possible if there are language barriers or other difficulties concerning clear communication (e. g. intoxication). If assessment is inadequate there is indication for complete immobilization. Situations that divert the patient’s attention, such as distracting injuries, states of anxiety as well as seriously injured or deceased relatives in an accident are all included under the term “serious distractions”. Assesment of the patient is limited by such distractions and the indication for complete immobilization (Fig. 1) is given. If assessment of the patient is not limited, factors that are associated with higher risk of spine injury can be evaluated (Table 4). These criteria are integrated into the E.M.S. IMMO Protocol as the MARSHAL criteria (Fig. 1). The MARSHAL criteria summarize the criteria mentioned in the current literature (Table 4) in a short and clear manner and are part of the E.M.S. IMMO Protocol. According to these criteria, full-body immobilization should take place if at least one of the MARSHAL criteria (midline spine tenderness; age ≥ 65 years; reduced sensibility or motor function; supraclavicular injuries; high speed accident (>100 km/h), MVA rollover, vehicle ejection; axial load to head, fall from ≥ 2 m; locomotive or bike collision) is confirmed. If all MARSHAL criteria can be positively excluded, the spine should be examined for pain or tenderness under manual pressure (Fig. 1). If this examination yields no pathological findings, the patient should be asked to actively turn the head 45° to both left and right side (Fig. 1). If this motion of the cervical spine is also possible without pain, immobilization is not necessary (Fig. 1).

The isolated use of a cervical collar does not provide adequate immobilization of the cervical spine [14–16] as described before. Nevertheless, cervical spine protection can be significantly improved by additionally immobilizing the trunk and extremities [15]. Complete immobilization can be achieved by also immobilizing the head [49]. Therefore, the E.M.S. IMMO Protocol does not distinguished between immobilization of the cervical spine and the remainder of the spine, since full-body immobilization should always be performed if there are indications of spine injury for the reasons given in the E.M.S. IMMO Protocol. Reduced immobilization using a cervical collar on its own and positioning in-line on the stretcher is only acceptable for patients in critical condition and with high priority for transport where ensuring rapid transport is essential (Fig. 1). According to the authors opinion, patients in stable conditions who were assessed for high risk of spinal injuries according to the E.M.S. IMMO Protocol should not be immobilized by a stand alone cervical collar.

### Applicability tests of the E.M.S. IMMO Protocol

**Fig. 3:**
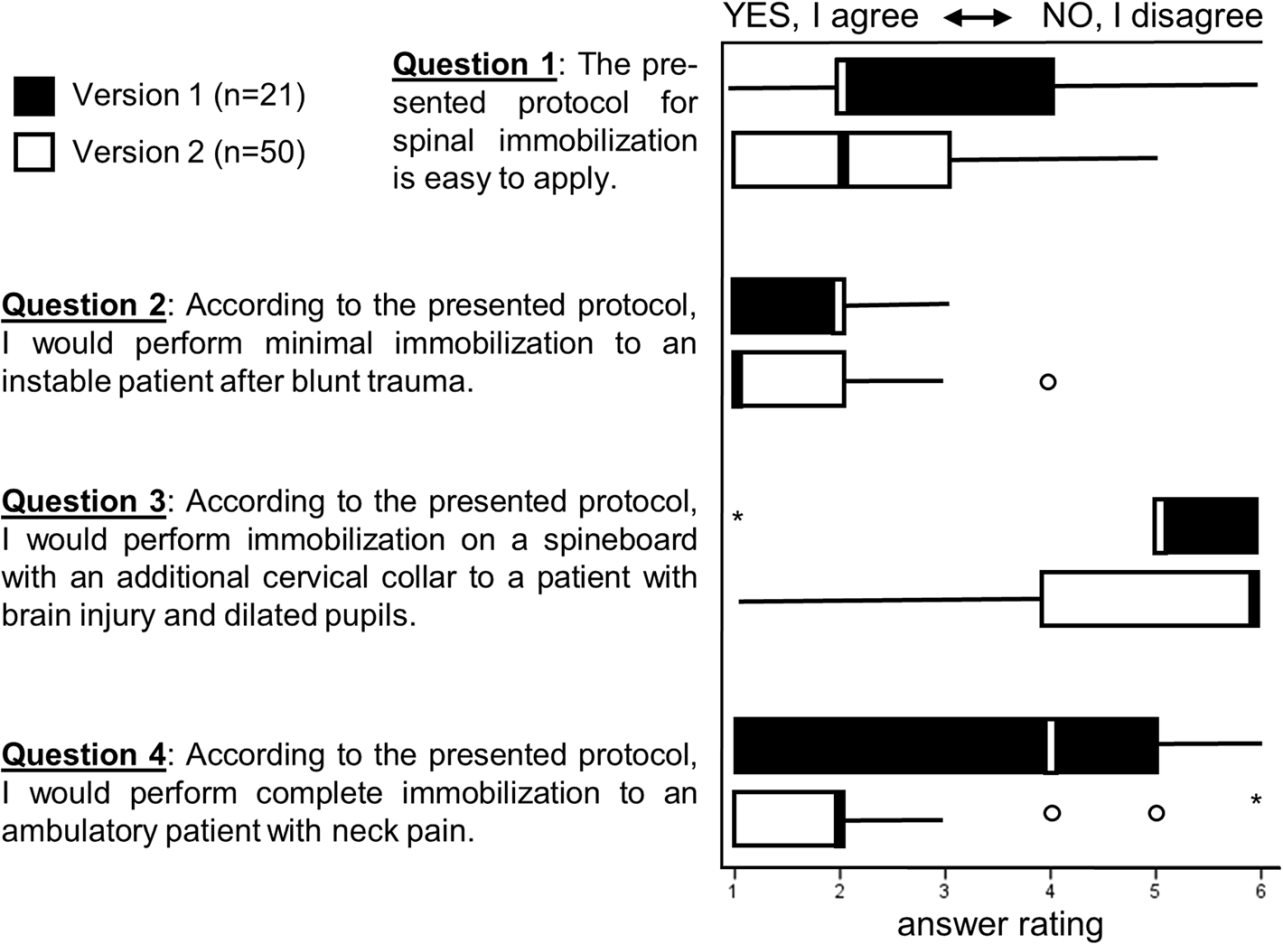
Analysis of the survey on applicability of the E.M.S. IMMO Protocol. The majority of participants agreed that the protocol was easy to use in both versions (Question 1). However, there was marked improvement in the responses to questions 2–4 regarding immobilization of trauma patients in the revised second version

In order to test the applicability of the E.M.S. IMMO Protocol, we planned to survey 50 German emergency medical care providers and emergency doctors. A first version of the E.M.S. IMMO Protocol was prepared (not shown). In the intermediate evaluation after *n* = 21 fully completed questionnaires, the consensus was that the E.M.S. IMMO Protocol was easy to apply (Fig. 3). However, questions 2–4 were frequently answered incorrectly (Fig. 3). The E.M.S. IMMO Protocol was therefore redesigned graphically to its current version (Fig. 1) since understanding problems seems to be based on graphic design.

The applicability of the current version of the E.M.S. IMMO Protocol (as shown in Fig. 1) was confirmed in a second survey of a different set of participants (*n* = 50). Furthermore, responses to questions 2–4 regarding immobilization of trauma patients were now improved (Fig. 3). In question 2 the median was reduced from 2 to 1 (interquartile range {1,2} in both cases) were 1 was considered the right answer. In question 3 the median was increased from 5 to 6 (interquartile range changed from {5,6} to {4,6}) were 6 was considered the right answer. In question 4 the median was reduced from 4 to 2 (interquartile range {1,5} reduced to {1,2} were 1 was considered the right answer (Fig. 3).

## Discussion

Based on the current literature and the requirements of the current guidelines, this study developed a protocol that supports decision-making for indications for out-of-hospital spinal immobilization. By integrating the decision-making process with the principles of the well known ABCDE principles for the care of trauma patients, we designed a dynamic protocol that orientates first on the current patient’s condition. Depending on the patient’s circulatory state, the protocol determines, for example, whether further evaluation of the spine is required or should be skipped because of high priority for transport. This should avoid immobilization procedures in unstable patients causing transport delay and thus increasing mortality. In contrast to established protocols [38, 50], the E.M.S. IMMO Protocol offers not only a decision-support tool to address whether immobilization is indicated or not, but also distinguishes between different options for immobilization. Hence, a patient with severe craniocerebral trauma and elevated intracranial pressure benefits from immobilization without a cervical collar, because this device may contribute to a further significant increase in intracranial pressure [20, 22]. To date, many protocols have considered the mechanisms of injury. Hence, there are specific protocols for penetrating [40–42] or blunt [43] trauma. The E.M.S. IMMO Protocol proposes no immobilization for unstable trauma patients following penetrating injury, because no benefits of immobilization have been demonstrated [33, 36, 43, 47, 48]. If a patient has suffered a penetrating injury with neurological symptoms but has stable circulation, which is assessed under C as well as D according to the ABCDE principles, full-body immobilization is performed based on the MARSHAL criteria.

There are increasing reports of disadvantages that may be associated with immobilization. For example, immobilization may cause restrictive effects on pulmonary function [23], airway management may be impeded [24, 25] and pressure ulcers [29] and pain [26–28] may result. Generalized full-body immobilization of every trauma patient is therefore not advocated by the E.M.S. IMMO Protocol. Application of the new formulated MARSHAL criteria and the subsequent examination of the spine result in differentiated indications for stable patients in order to avoid unnecessary immobilization but providing immobilization for those patient that really have a increased risk of spinal trauma. If a patient cannot be adequately assessed, complete immobilization should take place as a precaution.

The MARSHAL criteria were expressed for the E.M.S. IMMO Protocol on the basis of criteria from the recent literature. The majority of criteria that were formulated after comprehensive literature searches have been integrated into the E.M.S. IMMO Protocol. A few criteria were intentionally not adopted, as their wording was too generalized (e.g. road traffic collision, significant intrusion of vehicle, sport injuries). Other criteria are difficult to identify in a prehospital setting (e.g. other spine fractures) and were therefore not incorporated into the MARSHAL criteria. Criteria like high speed accidents are defined different in the current literature (>100 km/h vs. >56 km/h). Vaillancourt et al. could show that a threshold value of 100 km/h seems to be sufficient even for high sensitivity of an immobilization protocol [37, 51]. Therefore, this value was included into the E.M.S. IMMO Protocol.

One weakness of the study is the low number of participants in the first applicability tests. A multicentric study with a greater number of representative participants is required. In addition, a multicentric study should evaluate the sensitivity and specificity of the E.M.S. IMMO Protocol.

## Conclusions

The E.M.S. IMMO Protocol provides a new decision-support tool for indications for spinal immobilization in adult trauma patients that permits variable decision-making depending on the current condition of the trauma patient and the pattern of injuries. Furthermore, decision-support is given for different immobilization methods depending on the patient’s current condition and pattern of injury.

### Consent

Written informed consent was obtained from the person for the publication of the images.
